# Zinc and Propolis Reduces Cytotoxicity and Proliferation in Skin Fibroblast Cell Culture: Total Polyphenol Content and Antioxidant Capacity of Propolis

**DOI:** 10.1007/s12011-014-0019-3

**Published:** 2014-06-10

**Authors:** Małgorzata Tyszka-Czochara, Paweł Paśko, Witold Reczyński, Marek Szlósarczyk, Beata Bystrowska, Włodzimierz Opoka

**Affiliations:** 1 Department of Radioligands, Faculty of Pharmacy, Jagiellonian University Medical College, Medyczna 9, 30-688 Kraków, Poland; 2 Department of Food Chemistry and Nutrition, Faculty of Pharmacy, Jagiellonian University Medical College, Medyczna 9, 30-688 Kraków, Poland; 3 Department of Analytical Chemistry, Faculty of Material Science and Ceramics, AGH University of Science and Technology, Al. Mickiewicza 30, 30-059 Krakow, Poland; 4 Department of Inorganic Chemistry, Faculty of Pharmacy, Jagiellonian University Medical College, Medyczna 9, 30-688 Kraków, Poland; 5 Department of Toxicology, Faculty of Pharmacy, Jagiellonian University Medical College, Medyczna 9, 30-688 Kraków, Poland

**Keywords:** Zinc, Propolis, Fibroblasts, Cell viability, Wound healing, Oxidative stress

## Abstract

It has been demonstrated that zinc exerts its beneficial influence on skin fibroblasts. Propolis, a complex mixture of plant-derived and bees’ products, was reported to stimulate cicatrization processes in skin and prevent infections. The aim of this study was to find out how zinc and propolis influence human skin fibroblasts in cell culture and to compare the effect of individual compounds to the effect of a mixture of zinc and propolis. In this study, zinc, as zinc aspartate, at a concentration of 16 μM, increased human fibroblasts proliferation in cell culture, whereas propolis at a concentration of 0.01 % (*w*/*v*) revealed antiproliferative and cytotoxic action followed by mild cell necrosis. In culture, zinc was effectively transported into fibroblasts, and propolis inhibited the amount of zinc incorporated into the cells. An addition of propolis to the medium caused a decrease in the Zn(II) amount incorporated into fibroblasts. The obtained results also indicate an appreciable antioxidant property of propolis and revealed its potential as a supplement when applied at doses lower than 0.01 % (*w*/*v*). In conclusion, the present study showed that zinc had a protective effect on human cultured fibroblasts’ viability, although propolis revealed its antiproliferative action and caused mild necrosis.

## Introduction

Skin tissue protects the organism against pathogens and other external damaging factors. The proper functioning of this organ involves dynamic processes that, in turn, enable precise and accurate responses to environmental stimuli [1]. The dermal reconstruction after skin wounds depends on the effective coordination of all kinds of skin cell action, fibroblasts’ migration, and the precise regulation of inflammatory processes [2, 3]. In several reports, it has been indicated that zinc exerts its beneficial effect during wound healing [4–6]. In injured skin tissue, zinc takes part in the complex regulation of the sequence of signal molecules and mediators such as cytokines and growth factors, which then enable tissue regeneration [4, 7]. The mechanism involves the accurate cooperation of fibroblasts, platelets, endothelial, epithelial, and immune cells with the appropriate regulation and cross talk of signal transduction pathways in cells [2]. The skin’s ability to regenerate depends on biochemical processes regulated by zinc and, therefore, unbalanced homeostasis of this element affects basic cell functions. It was reported that in fibroblasts, zinc contributes to the decrease of reactive oxygen species’ (ROS) formation, since zinc metallothioneines (MTs) and Zn-Cu superoxide dismutase (SOD) neutralize highly reactive particles [8]. The activation of mechanisms against oxidative stress induced by zinc was demonstrated in several tissues [9]. As such, the protective effect also includes the aborted apoptosis of injured cells (zinc prevents the release of cytochrome C from mitochondria). Additionally, zinc acts as a suppressor of inflammation processes because it exerts an effect via the nuclear factor of the kappa-light-polypeptide gene enhancer in B-cells’ (NF-κB) signal transduction pathway [6].

A significant number of reports describe anti-inflammatory, immunomodulatory, antioxidant, anticancer, hepatoprotective, and many other biological activities of propolis [10–12]. Propolis, a substance produced by bees, contains numerous bioactive components, such as polyphenols [11, 13]. Therefore, along with the growing interest of naturally derived compounds, the pharmacological properties of propolis have been considered [13]. However, the advanced therapeutic use of this natural product is still limited according to its differences in chemical composition attributed to the specific botanical source and geographical region of origin, which themselves account for the different biological activities of area-distinct bee products [14, 15]. Thus, studies on propolis should include the determination of particular compounds such as polyphenolic contents, which have an influence on the antioxidant properties of this product. Notably, it is important to determine the possible detrimental effects such as cytotoxicity and the decreasing proliferation of a cell in target organ tissues.

Taken together, the estimation of interactions between compounds and revealing the aspects of their toxicity are critical steps during the evaluation process regarding drugs and their treatments, in order to avoid the unpredictable features during medical interventions. Defining the beneficial and adverse effects of zinc and propolis interactions on human fibroblast is a key step to avoid the unforeseen effects of therapies. Namely, the proper use of zinc and propolis in skin injuries might effectively help to improve the treatment of wound healing via the application of innovative wound dressings releasing bioactive particles.

The present study aims to (i) measure the concentration of zinc in cells after incubation without/with propolis and evaluate the possible influence of zinc transport into cells by propolis; (ii) estimate if zinc and/or propolis at established doses have any effect on the vitality of normal human skin fibroblasts; (iii) determine the antioxidant potential of propolis; and (iv) point out the aspects of the simultaneous use of both substances, zinc and propolis, in the cell culture of fibroblasts.

## Materials and Methods

### Materials

Eagle’s minimum essential medium (EMEM), fetal bovine serum (FBS), trypsin-0.05 % EDTA solution, and antibiotic solution were obtained from Gibco Laboratories (NY, USA). Phosphate-buffered saline (PBS) without Ca^2+^ and Mg^2+^, pH 7.4, were supplied by PAA Laboratories GmbH, Gotzis, Austria. Trypan blue, 3-[4,5-dimethylthiazol-2yl]-2,5-diphenyl tetrazolium bromide (MTT), hydrogen peroxide, zinc aspartate, bovine serum albumin (BSA), and dimethyl sulphoxide (DMSO) were purchased from Sigma-Aldrich, Seelze, Germany. Flow cytometry reagents were obtained from BD Biosciences, San Jose, CA, USA. All salts and other cell culture solutions were of cell culture grade (Gibco Laboratories, NY, USA). Sterile and non-toxic plates, flasks, tips, and centrifuge tubes were obtained from Sarstedt, Numbrecht, Germany. Reagents for quantitative determination of zinc were from Merck, Darmstadt, Germany. Antioxidant and polyphenol analyses’ reagents were from Sigma-Aldrich, Seelze, Germany. Raw pure propolis was purchased from Apipol Farma, Myslenice, Malopolskie, Poland. In the present study, a representative mixture of propolis obtained from apiaries located in North Poland was evaluated [16].

### Cell Culture

Human skin fibroblasts were derived from the American Type Cell Culture Collection (ATCC) (LGC Standards-ATCC (Teddington, UK), ATCC designation BJ, CRL-2522, normal adherent cells obtained from male, *Homo sapiens*). Fibroblasts were cultured in EMEM supplemented with 10 % *v*/*v* FBS and with antibiotic solution (100 IU/mL penicillin and 0.1 mg/mL streptomycin). The cells were kept at 37 °C in a humidified atmosphere of 5 % CO_2_. The cells used in the experiments were between 10th and 15th passages.

### The Studied Groups

The first group was a control one (C), where fibroblasts were incubated with adequate amounts of solvents. The second group (Zn) was incubated with 16 μM of zinc aspartate (10 μg Zn(II)/mL medium) [7, 17, 18]. Zinc aspartate was dissolved in buffered PBS, pH 7.4, and zinc solution was taken from 10 mM stock and added to the growth medium. The third group (P) was incubated with an addition of propolis at a concentration of 0.01 % (*w*/*v*). This was the maximum concentration of the compound, which would not precipitate in the culture media. The last one, the fourth group, Zn + P, was incubated in medium supplemented with 16 μM of zinc aspartate (10 μg Zn(II)/mL of medium) and 0.01 % (*w*/*v*) of propolis). All four of the studied groups were kept in the same medium and cell culture conditions.

### Incubation of Cells with Zinc and/or Propolis

After establishing the fibroblast culture, the medium was replaced with a new one also containing the tested compounds or the adequate amounts of solvents. As propolis contains trace elements [16], the concentration of Zn(II) in the medium with propolis was also measured (low but detectable 0.007 μg Zn(II)/mL medium). Incubation of cell culture supplemented with zinc and/or propolis was continued for 24 h. After incubation, the plates were examined by microscope (Olympus CKX 41SF-5, Olympus Optical Co., Ltd., Tokyo, Japan), and the cells were washed several times with buffered PBS, pH 7.4, to dispose the trace element in case it was adsorbed to the cell surfaces (the Zn(II) concentration was monitored in wash fluids). Then, the cells were centrifuged (150 × *g* for 10 min) and collected for zinc quantitative analysis and finally kept at −20 °C. The culture media at the beginning of the experiment and after 24 h of incubation were also collected to measure the concentration of Zn(II).

### Quantitative Determination of Zn(II) in Cells with Atomic Absorption Spectrometry

The quantitative determination of zinc in the cells, media, and wash fluid samples was made by a flame atomic absorption spectrometry (FAAS) using the Perkin Elmer spectrometer model 3110 (Perkin Elmer, Norwalk, CT, USA). The measurements were performed in an air-acetylene flame at 213.9 nm, slit 0.7 nm. A HCL single-element lamp was used. The samples were diluted appropriately to fit into the linear range of the calibration curve. In the case of samples of extremely low volume (with only a few microliters), the additive method of sample dilution was used. The concentration of zinc in the cells was determined using the slurry technique of sample nebulization. The cells, after refreezing, were suspended in ultrapure water and thoroughly mixed before nebulization.

The accuracy and precision of Zn quantitative determination were estimated based on the element determination in the test cell culture samples. Comparing the results obtained for the digested sample (Anton Parr Multiwave 3000 microwave system, wet digestion with 65 % HNO_3_ and 30 % H_2_O_2_; Suprapur®, Merck, Germany) and the untreated sample (slurry sampling), the recovery was repeatable and was 92 % (the samples were also spiked with the analyte). Precision of the measurements was similar for the preparation of both samples (RSD 4.5 % for the digested sample and 5.1 % for the untreated sample). All the glassware and equipment used in the analytical procedure was thoroughly washed with nitric acid and rinsed with quadruple distilled water. The standards and sample suspension were prepared using quadruple distilled water.

### Protein Concentration Measurement

The total protein amount in the cell samples was measured according to the Bradford method, with BSA as a standard and using a Universal Microplate Reader Bio-Tek ELX800NB (Bio-Tek Instruments, Inc., Vinooski, VT, USA).

### Cell Viability Assay (MTT)

For the MTT test, fibroblasts were seeded into 96-well plates at a density of 1 × 10^5^ cells/well in 200 μL medium. After 24 h, zinc and/or propolis solutions were added to media, and the incubation continued for the next 24 h. The control (100 % of growth) was cells cultured in medium and solvents only. At the end of incubation, the media were changed for new, containing additional MTT (5 mg/mL in PBS, pH 7.4). MTT formazan generated during incubation was dissolved in DMSO, and the absorbance was measured at 570 nm (the reference wavelength was 630 nm) using a Universal Microplate Reader Bio-Tek ELX800NB. For each sample, the result was expressed as a percentage of cells in the control [19].

### Live/Necrotic Cell Quantitation with Flow Cytometry

Fibroblasts were seeded into six-well plates at a density of 1 × 10^6^ cells/well in 2 mL medium. After 24 h, zinc and/or propolis solutions were added to media, and the incubation was continued for the next 24 h. Following the treatment, the cells were proceeded by live/necrosis quantifying, according to the manufacturer’s protocol (BD Biosciences, San Jose, CA, USA). Briefly, cells were harvested, washed twice with ice-cold PBS, pH 7.4, and centrifuged at 300 × *g* for 10 min. Cells were resuspended in binding buffer, and fluorochromes were added and incubated in the dark. Ethidium homodimer (EthD-III, with an excitation/emission of 528/617 nm) was used to measure the amount of necrotic cells in each sample. The fluorescence was excited by a laser and analyzed in LSRII flow cytometer, using FACSDiva software (BD Biosciences Immunocytometry Systems, San Jose, CA, USA). The cells were gated according to the forward (FSC), side scatter (SSC), and fluorescence parameters. The EthD-III negative fibroblasts were considered live cells, while EthD-III positive fibroblasts were accepted as the necrotic cells. The results were given as the percentage of the live cells of the total counted cells [19].

### Cell Colony Morphology

After 24 h of incubation, the morphology of fibroblast cell colonies was inspected with a phase-contrast microscope with a digital camera (Olympus CKX 41SF-5 microscope and CAM-UV 30 camera, Olympus Optical Co., Ltd., Tokyo, Japan). Proliferation as well as the hyperplastic changes in the cultures was evaluated in comparison to the control.

### Propolis Extract Preparation for Antioxidant Analysis

Samples of propolis (2 g) were extracted with 20 mL ethanol (96 %) for 3 h to prepare the ethanolic extract of propolis (EEP). The obtained extracts were decanted, centrifuged, and stored in darkness in a freezer at −24 °C. The solutions were later used for estimation of the total antioxidant activity (ferric reducing antioxidant power (FRAP) and free radical diphenylpicrylhydrazyl (DPPH) scavenging assay) and total phenol (TP) content.

### Determination of Total Phenols in Propolis Extract

The total of the phenols was determined colorimetrically using the Folin-Ciocalteau reagent, as described previously [17]. The total phenol assay was conducted by mixing 2.7 mL of deionized water, 0.3 mL of extracts, 0.3 mL 7 g/100 g Na_2_CO_3_, and 0.15 mL Folin-Ciocalteu reagent. Absorbance of the mixture was measured at 725 and 760 nm using the spectrophotometer Jasco UV/Vis-530 (Jasco International Co., Ltd., Tokyo, Japan). A standard curve was prepared with gallic acid. The final results were given as gallic acid equivalents (GAE).

### The Total Antioxidant Activity of the Propolis Extract

#### FRAP Method

The FRAP assay was carried out according to Benzie and Strain [20] and modified to 48-well plates and an automatic reader (Synergy-2, Bio-Tek Instruments, Inc., Vinooski, VT, USA) with syringe rapid dispensers. The FRAP assay was conducted at 37 °C and pH 3.6. Ferric (Fe^3+^) to ferrous (Fe^2+^) ion reduction causes the formation of an intensive blue-colored ferrous-tripyridyl-s-triazine complex with an absorbance maximum at 593 nm. The absorbance was measured after 15 min and was proportional to the combined ferric reducing/antioxidant power of the antioxidants in the extracts. The final results were expressed as mmol Fe^2+^/g of the dry weight.

#### The DPPH Method

The radical scavenging activity of the propolis samples against DPPH was measured according to Davalos et al. [21], with a modification as follows. One milliliter of methanolic DPPH solution (25 mg/L) was mixed with 25 μL of propolis solution. The mixture was shaken and left in the dark at 30 °C for 24 h, and the absorbance was recorded at 517 nm. The antioxidant activity was measured using the spectrophotometer Jasco UV-530 as the percentage of DPPH (%DPPH) remaining in the solution with respect to the control values.

#### Statistical Analysis

Statistical analysis of cell culture experiments was performed by a one-way ANOVA with Bonferroni’s multiple comparison posttest, which was performed using the GraphPad Prism version 5.00 for Windows, GraphPad Software, San Diego, CA, USA. The results of the analyses of antioxidant activity were given as means ± SD, based on four measurements for each sample; the results of zinc concentration were given as means ± SD, based on three experiments.

## Results

### Concentration of Zinc in Cultured Human Fibroblasts After Incubation with Zinc or/and Propolis

The concentrations of Zn(II) in human fibroblasts for all experimental groups are demonstrated in Table 1. The results show that Zn(II) concentration in the Zn group was significantly higher than that in the control (*p* < 0.001) and significantly lower in the P group when compared with the control (*p* < 0.001). The highest concentration of the element was measured in cells from the Zn group, and the lowest in cells from the P group (with significant differences between the Zn group and P group at *p* < 0.001). The treatment of cells with zinc and propolis caused the Zn(II) concentration in the Zn + P group to be significantly lower than in the Zn group (*p* < 0.001) and higher than in the P group (*p* < 0.001), with no difference between Zn + P and control groups (*p* < 0.001).

**Table 1:** Zn(II) concentration in a human fibroblast after 24 h of incubation with estimated amounts of compounds

Experimental group	Zn(II) concentration in cells (μg/g of total protein) after 24 h of incubation
Control (C)	0.076 ± 0.002^a^
Zinc (Zn)	1.132 ± 0.057^b^
Propolis (P)	0.030 ± 0.002^c^
Zinc + Propolis (Zn + P)	0.067 ± 0.002^a^

The values were expressed as a mean percentage with standard deviation. The mean values with different subscript letters were significantly different (*p* < 0.001), *n* = 3

### Effect of Zinc or/and Propolis on Human Fibroblast Proliferation

**Fig. 1:**
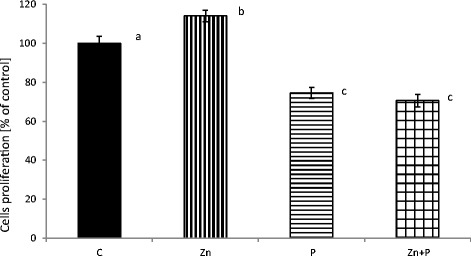
Effect of zinc and propolis on normal human fibroblast proliferation. Cell viability was measured by the MTT test after 24 h of incubation with estimated amounts of compounds. The values were expressed as a mean percentage with standard deviation. The *mean values with different subscript letters* were significantly different (*p* < 0.001), *n* = 6

The effect of 16 μM zinc in the presence of 0.01 % (*w*/*v*) propolis on human fibroblasts’ viability, following 24 h of incubation, is demonstrated in Fig. 1. The highest cell proliferation was measured after incubation with zinc aspartate (with significant differences compared with the control at *p* < 0.001). Following incubation of fibroblasts with an addition of propolis, and zinc and propolis together, a significant decrease in the cells’ viability was observed when compared with the control and zinc groups (with all the differences significant at *p* < 0.001).

### Live/Necrotic Cell Quantitation Using Flow Cytometry

**Fig. 2:**
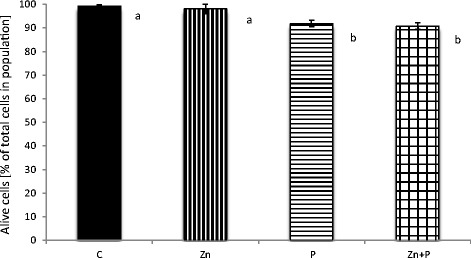
Effect of zinc and propolis on normal human fibroblasts vitality. The decrease in the percentage of live cells after 24 h of incubation was due to cell necrosis measured as the changes in the fluorescence of ethidium homodimer with flow cytometry. The values were expressed as a mean percentage with standard deviation. The *mean values with different subscript letters* were significantly different (*p* < 0.001), *n* = 6

The effect of zinc and/or propolis on human fibroblasts’ vitality is demonstrated in Fig. 2. The scale bars represent the percentage of live cells after incubation. The decrease in the number of live cells in the appropriate samples when compared with the control was due to necrosis. No disrupting effect on cells incubated with an addition of zinc when compared with the control was observed (with no significant differences between these groups at *p* < 0.001). The incubation of cells with propolis, and with zinc and propolis together, caused a significant decrease of the cells’ vitality when compared with the control (with significant differences at *p* < 0.001). There were no significant differences between groups P and Zn + P at *p* < 0.001.

### Changes in the Morphology of Culture of Human Fibroblasts after Incubation with Zinc or/and Propolis

**Fig. 3:**
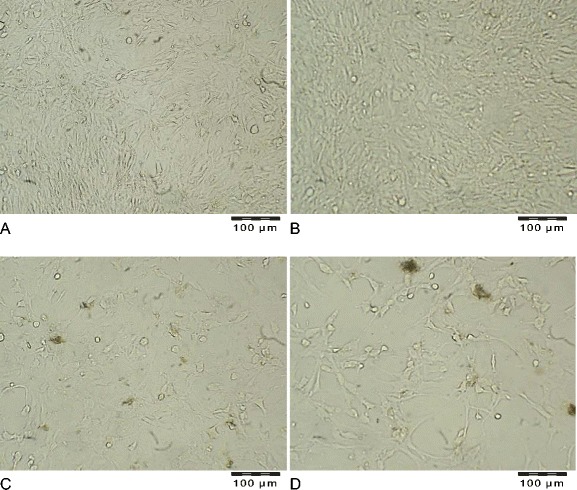
Representative images of human fibroblasts incubated for 24 h with the addition of. **a** adequate amounts of solvents (group C–control), **b** 0.16 mM of zinc aspartate (10 μg Zn(II)/mL medium) (group Zn), **c** propolis at concentration 0.01 % (*w*/*v*) (group P), **d** 0.16 mM of zinc aspartate (10 μg Zn(II)/mL medium) and 0.01 % (*w*/*v*) of propolis (group Zn + P). Magnification × 250

Microscopic analysis of fibroblasts incubated with zinc revealed that the cells did not change the typical spindle shape. A higher cell density was observed in the Zn group (Fig. 3b) when compared with the control (Fig. 3a). The addition of propolis to culture media caused an evident loss in the number of cells (Fig. 3c). A low proliferation ratio was also observed in the culture with an addition of both zinc and propolis (Fig. 3d). The demonstrated decrease in the number of cells (Fig. 3c, d) when compared with the control (Fig. 3a) were followed by differences in cell shape, which changed from being elongated and mutually aligned in the control (Fig. 3a) and Zn groups (Fig. 3b) to being less regularly adhered, with a tendency to form aggregates and even an agglomerated growth type (Fig. 3d).

### The Polyphenol Content and the Antioxidant Activity of Propolis Extract

**Fig. 4:**
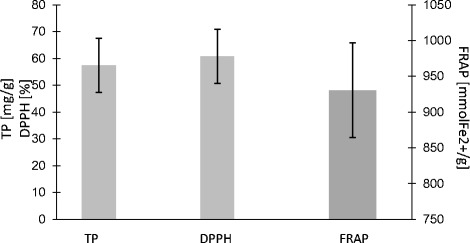
Total polyphenol content, TP (mg/g) and antioxidant capacity (ferric reducing antioxidant power, FRAP mmol Fe^2+^/g, and free radical diphenylpicrylhydrazyl scavenging assay, %DPPH) of ethanolic extracts of propolis (EEP). The values were expressed as a mean percentage standard deviation, *n* = 4

The antioxidant parameters are demonstrated in Fig. 4. The total phenolic content of propolis was 57.40 ± 8.60 mg/g, and the antioxidant capacity measured using two methods (FRAP and DPPH) showed activity as follows: 930.5±66.34 mmol Fe^2+^/g and 60.78±10.12 %. The antioxidant status parameters FRAP, DPPH, and TP were positively correlated. However, there were strong correlations between the results of DPPH versus FRAP (*r* = 0.88; *p* < 0.05), TP versus FRAP (*r* = 0.95; *p* < 0.05), and TP versus DPPH (*r* = 0.97; *p* < 0.05).

## Discussion

The idea of using naturally occurring substances in medical treatment and complementary and alternative therapies has recently become more popular [22]. The human skin fibroblasts used in the presented experiments are a reliable tool for screening tests in hazard assessment. Regarding the criteria for cell culture models, fibroblasts constitute a non-tumor human cell line with the regulation of metabolic processes comparable to pathways in skin tissue cells. In order to investigate the possible biological interactions of zinc and propolis when applied together, we performed experiments in a culture of normal human fibroblasts. In the present study, it was shown that the amount of zinc transported into fibroblasts after incubations with propolis was significantly lower in cells incubated only with 16 μM zinc and also lower in the control cells (Table 1). The data indicates that propolis influenced the transportation process of the element into cells. This finding is consistent with the results obtained in the viability test. In this study, the MTT quantitative colorimetric test was used to assess the influence of zinc and propolis on cells in the culture measured as changes in cells mitochondrial succinate dehydrogenases activities. The incubation of fibroblasts with zinc and propolis together caused a decrease in the number of viable cells compared with the control, as well as when compared with cells incubated only with zinc (Fig. 1). The adverse effect of 0.01 % (*w*/*v*) propolis (alone and together with 16 μM zinc) on the proliferation of human fibroblasts was followed by a decrease of live cells and a concomitant increase of necrotic cells in the population measured with the flow cytometry assay (Fig. 2), plus also by the changes in the cell morphology, because the microscopic observations revealed that fibroblasts showed deviations from regular, spindle-shaped forms (Fig. 3c, d). As the morphology of eucaryotic cells is closely related to their function, the observations of cell cultures confirmed the cytotoxic and anti-proliferative effect of propolis on fibroblasts. The reported studies concerning the effect on fibroblasts included its high [10] or mild cytotoxicity [23], no effect [24], and even the pro-proliferative activity of this product [25], depending on concentration, time of exposure, and the in vitro/in vivo conditions. Funari et al. [10] reported that concentration of 31.25 μg/mL of propolis extract (containing 7.39 % *w*/*w* of total polyphenols) was toxic to mouse fibroblasts and caused about a 50 % decrease in cell viability. In the presented study, propolis at a concentration of 100 μg/mL, containing 57.40 ± 8.6 mg/g of total polyphenols, decreased cell growth in 27 % when compared with the control. Sobocanecet al. [26] pointed out that propolis components may demonstrate not antioxidant but pro-oxidant properties, depending on the experimental conditions, and at high concentrations, propolis exerts rather adverse than beneficial effects on cells. The obtained data confirmed that it is essential to apply the proper amount of propolis to reveal the protective influence of propolis polyphenols on cells without inducing cellular stress.

The relevance of application of zinc and its protective effect on human cells may depend on its antioxidant activity. Recently, zinc’s action on a molecular level was intensively investigated [27]. It has been reported before that zinc influences the function of cells, promotes cell proliferation and differentiation, and acts as a protective factor [28]. In our experiments, zinc at a concentration of 16 μM enhanced fibroblasts’ viability and proliferation (Fig. 1). Incubation of fibroblasts with this element at a concentration of 160 μM (data not shown) caused an apparent decrease of fibroblasts’ proliferation (only 20 % of the cell population was alive after 24 h of incubation). The addition of propolis at 0.01 % (*w*/*v*) to the culture medium containing zinc (160 μM) caused enhanced cytotoxicity, and the percentage of living cells was only 5 %, which corresponds to the trend observed in the presented experiments. Notably, our data confirmed that zinc at a concentration of 16 μM, tenfold the average value in human serum [18], was effectively incorporated into cells (Table 1) and, acting inside the cells, caused a significant increase in fibroblasts’ proliferation rate. This was the prospective effect, as fibroblasts play a crucial role in the formation of scar. On the other hand, the addition of zinc to 1.6 μM medium (the average concentration of the element in human serum) had no effect on the fibroblasts’ performance (data not shown). Zinc transport into and inside the cells involves two families of mammalian-specific zinc transporters: ZIP/SLC39 family (Zrt- and Irt-like proteins, solute-linked carrier 39 (SLC39)), which transport zinc into the cytoplasm and ZnT/CDF/SLC30 family transporter (ZnT/CDF zinc transporter, solute-linked carrier 30 (SLC30)), which then cause the efflux of zinc ions from the cell [8]. Propolis compounds might influence these proteins’ action, and that way induced changes in the zinc transport ratio. Another aspect to be considered was the possible chemical interactions between propolis components and zinc in cell medium, which might lead to a reduced accessibility of the element for cell transporters.

The antioxidant activity of propolis extract results mainly from the content of polyphenols. It is known that various plant extracts abounding in antioxidants are useful in the prevention or treatment of skin disorders, especially those mediated by infectious agents and irradiation. ROS can cause harmful effects in cells, especially when the intrinsic antioxidative defense mechanisms are exhausted [29]. A lot of natural compounds present in propolis had been tested alone or in combinations for the prevention of sunburn, photodermatoses, and photocarcinogenesis, with encouraging results [30]. The antibacterial action of bees’ products was confirmed several times, even against methicyllin-resistant *Staphylococcus aureus* (MRSA) in clinical use, when the decreased risk of infections due to the debriding effect was observed [31]. A lot of natural products were considered as potential agents in wound healing, and this kind of “natural therapy” is currently preferred because of its widespread availability, the ease of administration, and its effectiveness, plus, most of all, because of the common belief that they are non-toxic to the skin cells. However, it should be emphasized that propolis extracts at high doses may cause a harmful effect in normal mammalian cells [10].

The pharmacological activity of propolis depends strongly on its origin, and therefore, the evaluation of specific compounds is highly required. Polyphenols were pointed out to be the most responsible for important biological properties [32]. Antioxidant parameters such as FRAP, DPPH, and TP are strongly associated with the amount and potency of polyphenols. In the present study, we have found that Polish propolis had strong DPPH free radical scavenging activity of over 60 %, similar to a product that originated in China [15] (Fig. 4). When considering FRAP activity, a precise indicator of antioxidant capacity, Polish propolis extract demonstrated average values [33]. According to Ahn et al. [34], the presence of such polyphenols as kaempferol, quercetin, and caffeic acid phenethyl ester in propolis is associated with a strong antioxidant capacity. The total phenolic content in the tested Polish propolis was low (57.40 ± 8.60 mg/g of GAE) when compared with samples from other countries, but the specific composition of Polish propolis [16] apparently influenced its high activity toward free radicals.

In conclusion, zinc derived from the organic compound, zinc aspartate, at a concentration of 16 μM, had a beneficial effect on growth, proliferation, and metabolism of normal human skin fibroblasts. The performance of zinc was perturbed by the addition of 0.01 % (*w*/*v*) propolis to the culture medium, which decreased the incorporation of this trace element into cells and/or made zinc less accessible to fibroblasts. Due to its activity, zinc is used in the daily treatment of numerous kinds of skin wounds as a part of pharmaceutical formulations (ointments, creams, pastes, gels, and powders) and also as an oral supplementation recommended for patients with a long-term ineffectual therapy of wounds. Propolis, which is very popular in traditional and folk medicine, seems to be a preferable agent in the prevention of wound infections and efficiently inhibits the spread of the surface of contamination. Unfortunately, in our in vitro study, interactions between zinc and propolis were harmful for skin fibroblast performance due to the adverse effects on fibroblast vitality during the application of zinc and propolis together. The obtained results revealed new aspects of interactions between zinc and propolis in vitro conditions and suggest the need to continue the studies to assess the outcomes of the interplay between this element and apicultural medical products in one drug’s delivery system.
